# Findings From Somatic and Cerebral Near-Infrared Spectroscopy and Echocardiographic Monitoring During Ductus Arteriosus Ligation: Description of Two Cases and Review of Literature

**DOI:** 10.3389/fped.2020.00523

**Published:** 2020-09-02

**Authors:** Carolina Michel-Macías, Deneb Algedi Morales-Barquet, Alfonso Martínez-García, Daniel Ibarra-Ríos

**Affiliations:** ^1^Instituto Nacional de Perinatología (INPER), Mexico City, Mexico; ^2^Hospital Infantil de México Federico Gómez, Mexico City, Mexico

**Keywords:** NIRS (near infrared spectroscopy), ductus arteriosus, tissue oxygenation, postligation cardiac syndrome, cerebral oxygenation, multisite NIRS, somatic-cerebral difference, HsPDA (hemodynamically significant patent ductus arteriosus)

## Abstract

**Background:** Preterm infants with hemodynamically significant patent ductus arteriosus (HsPDA) are exposed to low cerebral tissue oxygen saturation (rScO_2_) values. Additionally, infants requiring surgical ligation are at risk of further changes in cerebral oxygenation and postligation cardiac syndrome (PLCS). Previous studies have assessed the effect of PDA ligation on rScO_2_ with variable results.

**Cases description:** In this report we analyse near-infrared spectroscopy (NIRS) and echocardiographic findings of two patients who underwent ligation of PDA and presented low cardiac output. Literature on regional tissue oxygenation saturation (rSO2) before and after PDA ligation was briefly reviewed.

**Discussion:** Cerebral oxygenation values before and after PDA ligation may be influenced by gestational age, vasopressor use, ductal shunt volume, time of exposure HsPDA, chronological age and the presence of cerebral autoregulation. PLCS complicates 28–45% of all PDA ligations and is associated with higher mortality. Cerebral and somatic NIRS monitoring in the postoperative period may enhance the identification of PLCS at early stages.

**Conclusion:** Cerebral oxygenation in the perioperative period of PDA ligation may be influenced by numerous clinical factors. Early detection of PLCS using multisite NIRS after ligation could prevent further alterations in cerebral hemodynamics and improve outcomes. A decrease in somatic-cerebral difference and/or a significant drop in somatic NIRS values may precede clinical signs of hypoperfusion. NIRS values should be interpreted as trends along with echocardiographic findings to guide goal directed interventions.

## Background

Preterm infants with HsPDA are exposed to low rScO_2_ which poses a risk of cerebral injury (1, 2). As shown using pulsed-wave Doppler, HsPDA may negatively influence brain perfusion. Furthermore, a transient additional reduction in cerebral oxygenation occurs during ductal closure surgery (1). An increased risk of neurosensory impairment has been reported in infants who underwent PDA surgical closure (1). Previous studies have reported differing results regarding cerebral tissue oxygenation after PDA ligation. Renal tissue oxygen saturation (rSrO_2_) values have not been monitored during surgical ligation of ductus arteriosus but have been reported to normalize after closure with indomethacin and ibuprofen (3–5).

PLCS complicates 28–45% of all PDA ligations. It is more frequent in the youngest and most immature infants and has been shown to increase mortality (6, 7). Near-infrared spectroscopy (NIRS) is a non-invasive method to measure tissue oxygenation and perfusion in regions of interest (8). Previous studies demonstrated a correlation between somatic NIRS and indirect measures of CO such as mixed venous saturation and lactate levels in infants with congenital heart disease (CHD) (9). Monitoring rScO_2_ and rSrO_2_ after PDA ligation could provide valuable information for early detection of poor perfusion in critical organs.

Herein we report the evolution of cerebral and renal tissue oxygenation as well as left ventricular cardiac output in two patients with a HsPDA before, during and after surgical ligation. To our knowledge, this is the first report to describe continuous cerebral and somatic tissue oxygenation during PLCS.

## Case 1

A 30 week gestation, 1,255 g male infant was born by vaginal delivery to a 22 year old mother. Betametasone and magnesium sulfate were administered 24 h before birth. The infant was born non-vigorous and persisted bradycardic after 60 s of positive pressure ventilation, for which was intubated. Apgar score was 3/8 at 1 and 5 min, respectively. FiO_2_ requirements reached 100% and surfactant was administered. He was extubated to CPAP on the second day of life.

**Figure 1:**
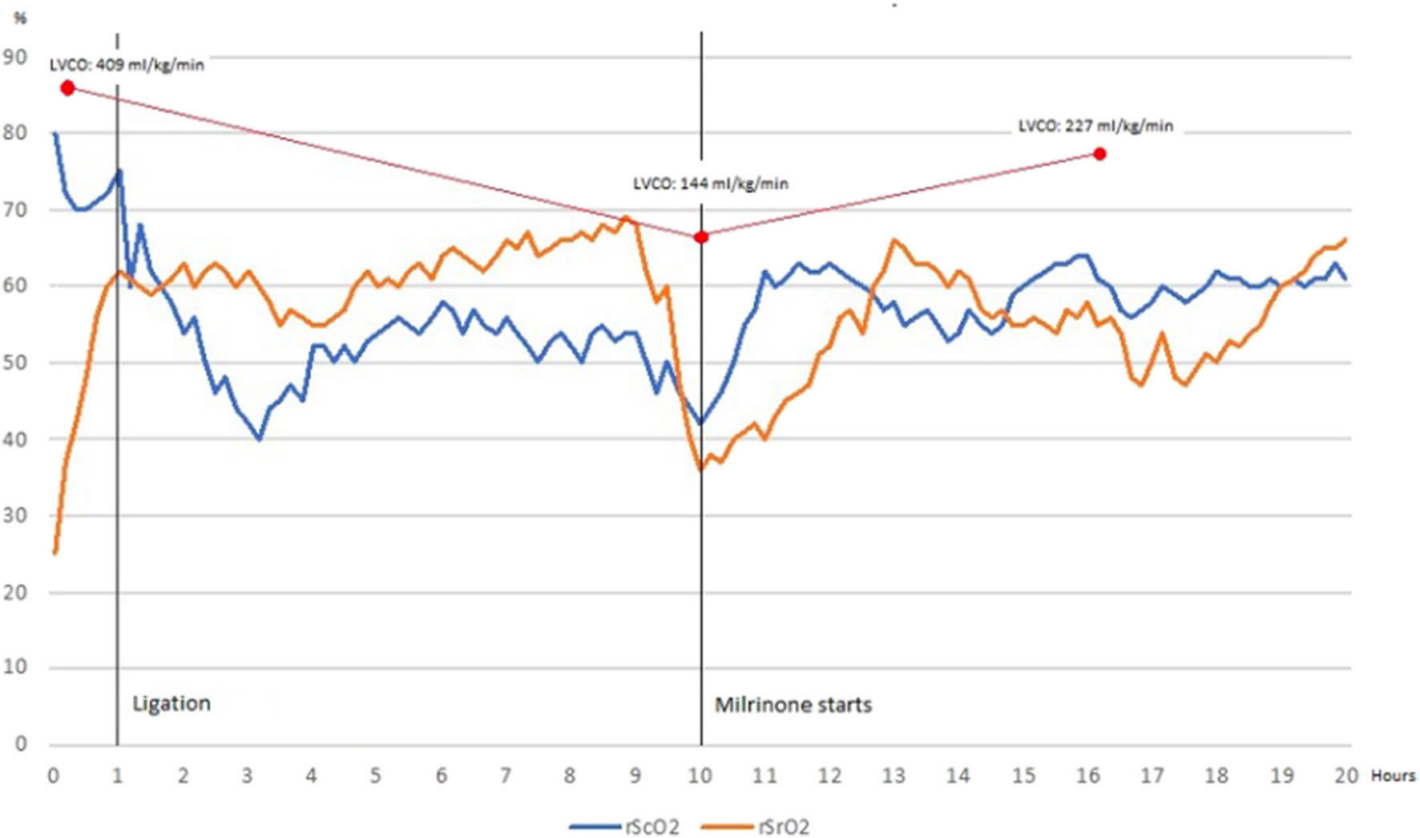
Cerebral tissue oxygenation declines and renal tissue oxygenation increases after ductus ligation to reach a normal somatic-cerebral difference, which is maintained 9 h before rSrO_2_ decreases significantly and somatic-cerebral difference is inverted (blood flow redistribution). Once Milrinone is started, rSrO_2_ recovers as well as somatic-cerebral difference. When monitoring finishes, renal and cerebral tissue oxygenation are normal and somatic-cerebral difference is preserved.

Echocardiography on day 5 of life revealed a hemodynamically nonsignificant patent ductus arteriosus for which spontaneous closure was expected. The criteria for determining an HsPDA were used as previously described by McNamara et al. (10). Echocardiographic reassessment on day 40 of life due to mechanical ventilation dependency revealed a HsPDA with reverse end-diastolic flow in the abdominal aorta but surgical ligation was withheld due to gastrointestinal bleeding. Diuretic therapy was started. On day 58 of life, after GI bleeding remission, surgical ligation was performed and monitoring with NIRS was implemented and registered prior, during surgical ligation and on the first 20 postsurgical hours. Baseline rScO_2_ and rSrO_2_ were 79 and 25%, respectively. RScO_2_ presented an expected drop after ligation. After a steady increase, rScO_2_ and rSrO_2_ decreased significantly 10 h after ligation. No hypotension, oliguria or delayed capillary refill were present. Echocardiographic evaluation showed low cardiac output and left ventricular dysfunction. Milrinone was started and maintained for 24 h. Ventricular function improved and normalized afterwards and NIRS values increased progressively toward normal values. Echocardiographic and NIRS findings are shown in Table 1 and Figure 1.

**Table 1:** Echocardiographic and NIRS findings.

**Day of life**	**40**	**51**	58 (PDA Ligation)		
PDA diameter (mm)	3.4	3.2			
PDA maximum gradient (mmHg)		33			
LA/Ao		2.5			
E/A		0.9			
IVRT (ms)		34	Preligation	9 h^a^	20 h^b^
LVCO (ml/kg/min)		409	144	227	
LVEF (%)			41	66	
Abdominal Aorta	Reverse end-diastolic flow	Reverse end-diastolic flow			
rScO_2_ (%)			79	42	60
rSrO_2_ (%)			25	37	56

*PDA, patent ductus arteriosus; LA/Ao ratio, Left atrial to aortic ratio; E/A, Mitral E/A waves ratio*.

*IVRT, Isovolumic relaxation time; LVCO, left ventricular cardiac output; LVEF, left ventricular ejection fraction*.

rSrO_2_, regional renal oxygen saturation; rScO_2_, regional cerebral oxygen saturation.

*Preligation values*.

a 9 h postligation;

b *20 h postligation*.

## Case 2

A 28 week gestation, 920 g female infant was born by cesarean section to a 30 year old mother with chronic kidney disease and uncontrolled secondary systemic arterial hypertension. Methylprednisolone had been administered previously as a part of her chronic kidney disease management. Magnesium sulfate was administered 24 h before birth. The infant was born non-vigorous and required 60 s of positive pressure ventilation. She was intubated and surfactant was administered. Apgar score was 3/8 at 1 and 5 min, respectively. After 2 h she was extubated and placed in nasal ventilation.

**Figure 2:**
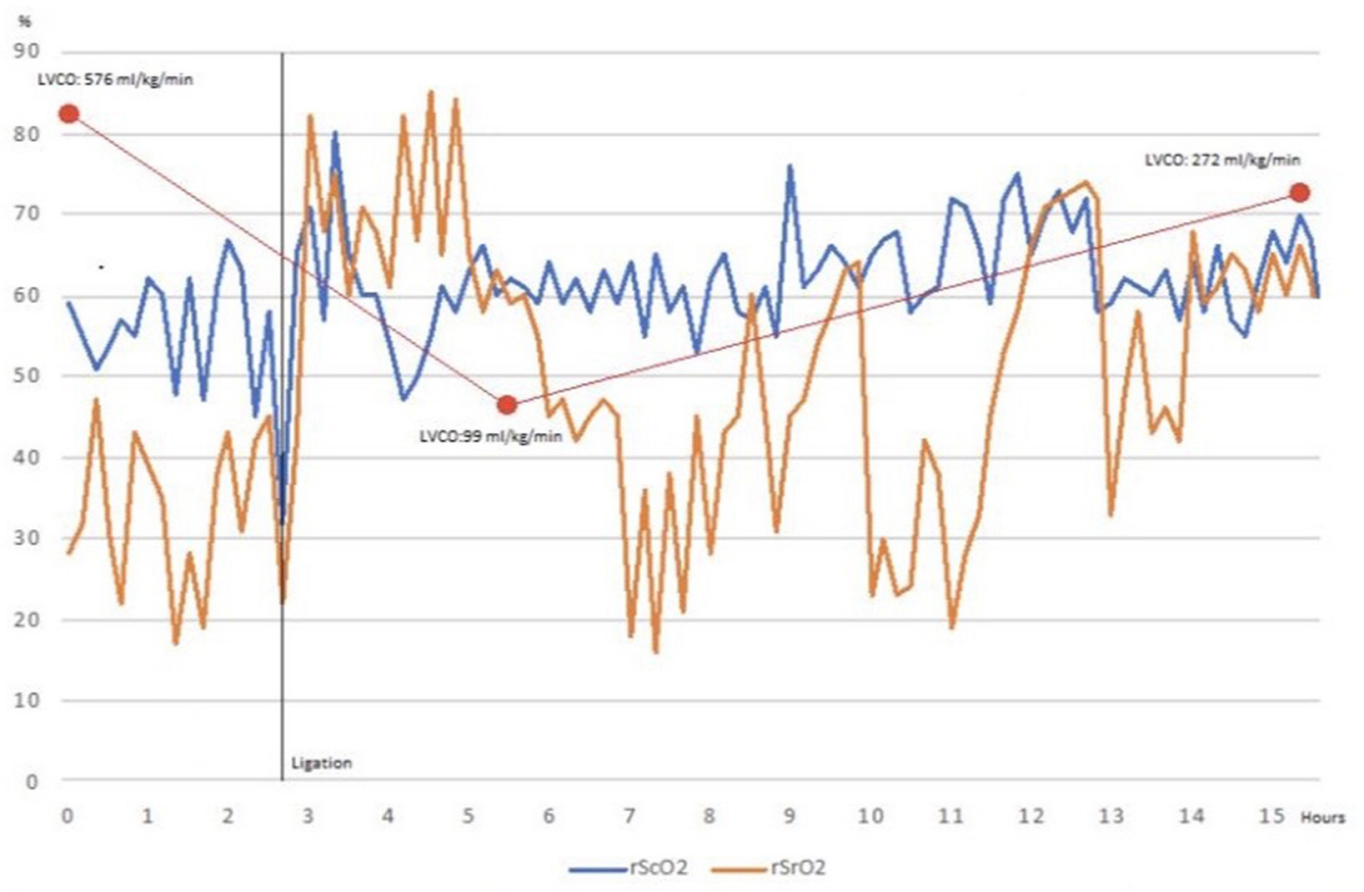
After ligation, cerebral and renal tissue oxygenation increase. Echocardiographic reassessment (which coincides with an inverted somatic-cerebral difference) reveals low LVCO. After Milrinone starts renal tissue oxygenation increases slowly. Somatic-cerebral difference is preserved when echocardiographic re-evaluation shows a normal LVCO.

Echocardiographic assessment on day 5 of life showed a moderate-volume shunt HsPDA. Paracetamol was administered at 15 mg/kg/dose for 5 days. On day 9 of life she presented diastolic hypotension and oliguria for which dopamine was started at 10 μg/kg/min. Echocardiographic reevaluation revealed a 1.8 mm diameter PDA and second cycle of paracetamol was started. Vasopressor was suspended on day 10 of life and the patient was placed on CPAP with FiO_2_ 30%. Because of oxygen dependency, an echocardiographic reassessment was performed on day 19 of life showing left side dilation and absent diastolic flow on celiac trunk. A high dose cycle of ibuprofen was started. A subsequent echocardiogram on day 24 of life while continuing on CPAP showed an increasing diameter of PDA and reversed diastolic flow on celiac trunk. Surgical ligation was indicated and performed on day 26 of life. NIRS monitoring was placed 2 h before surgery and maintained for 14 h. Baseline rScO_2_ and rSrO_2_ were 59 and 28%, respectively. Both NIRS values decreased during ligation and increased considerably afterwards. One hour after surgical procedure, echocardiographic evaluation was performed according to recent guidelines (6). Due to LVCO, a crystalloid load was administered at 10 ml/kg and milrinone was started at 0.33 μg/kg/min. A subsequent echocardiogram showed normal LVCO. At the end of recording, NIRS values trend toward normalization (Table 2 and Figure 2).

**Table 2:** Echocardiographic and NIRS findings.

**Day of life**	**5**	**9**	**19**	**24**	26 (PDA Ligation)		
PDA diameter (mm)	3.2	1.8	3.2	3.8			
PDA maximum gradient (mmHg)		34	31	75			
LA/Ao ratio	1.8	1.27	2.0				
E/A ratio	0.7	0.8	0.8				
IVRT (ms)					Preligation	1 h^a^	12 h^b^
LVCO (ml/kg/min)	392.5		443	576	576	99	272
LVEF (%)			41			38	40
SF (%)						16.9	18.1
LVDD (mm)						14.8	14.5
Abdominal Aorta	Absent end-diastolic flow	Absent diastolic flow		Reversed diastolic flow			
rScO_2_ (%)					59	62	57
rSrO_2_ (%)					28	60	64

*PDA, patent ductus arteriosus; LA/Ao ratio, Left atrial to aortic ratio; E/A, Mitral E/A waves ratio; IVRT, Isovolumic relaxation time; LVCO, left ventricular cardiac output; LVEF, left ventricular ejection fraction; SF, Shortening fraction; LVDD, Left ventricular diastolic diameter*.

*rSrO_2_, regional renal oxygen saturation; rScO_2_, regional cerebral oxygen saturation*.

a 1 h postligation;

b *12 h postligation*.

## Discussion

Previous studies have assessed the effect of PDA closure on cerebral oxygenation values with variable results. Surgical ligation of PDA has resulted in increased, decreased or unvaried cerebral oxygenation compared to baseline values [Supplementary Table 1; (3, 4, 11–16)]. These conflicting results suggest that cerebral oxygenation values before and after ductal closure may be influenced by gestational age, ductal shunt volume, time of exposure to ductal shunt, chronological age and the presence of cerebral autoregulation, as well as vasopressor use and previous pharmacological treatment.

Cerebral oxygenation values recorded for brief periods or in certain points in time may provide misleading information in some cases. Chock et al. reported that infants who had their HsPDA surgically ligated were more likely to have had significant changes from their baseline cerebral oxygenation compared to those treated with indomethacin or conservative treatment (12). Transient cerebral hypoxemia after surgical ligation of PDA could result from reperfusion or increasing perfusion of different cerebral zones due to changes in vascular tone preceding compensatory effects of increased cardiac output (17).

As PLCS is associated with higher mortality and increased risk for severe bronchopulmonary dysplasia (BPD), the use of strategies to detect this syndrome at early stages (before end organ hypoperfusion) can lead to improved outcomes in preterm infants with PDA (6, 7).

The NIRS measures of rSO2, although inherently imprecise, have the advantage of continuous and noninvasive availability, with adequate precision to detect clinical risk conditions. Multisite rSO2 measurements may be a superior methodology to detect organ hypoxia-ischemia (9). Cerebral oxygen extraction (~20%) is higher than renal oxygen extraction (~10%). This finding suggests that normal arterioregional differences could also be target for circulatory management. Hoffman et al. reported that somatic-cerebral rSO2 difference < 10 highly correlated to anaerobic metabolism (*p* < 0.001) (9).

In the aforementioned cases, rSrO_2_ baseline values were abnormally low due to reversed diastolic flow in descending aorta. After PDA ligation, a significant reduction (>20%) in rSrO_2_ and somatic-cerebral difference concurred with low LVCO in both cases in the absence of hypotension, oliguria and/or delayed capillary refill. In case 1, cerebral oxygenation decreased significantly at the end of measurement period compared to preligation baseline values (60 vs. 80%). In case 2, rScO_2_ remained unvaried.

Early detection of PLCS using multisite NIRS after ligation could prevent further alterations in cerebral hemodynamics and improve outcomes. A decrease in somatic-cerebral difference and/or a significant drop in somatic NIRS values may precede clinical signs of hypoperfusion. Further studies are needed to investigate the applicability of somatic-cerebral difference in preterm infants. Our report has many weaknesses. Firstly, we describe only two cases and no conclusions can be drawn regarding our findings. Secondly, somatic-cerebral difference although physiologically founded, has not been used in preterm infants. We consider that continuous and long lasting NIRS recording is a strength of this report, as well as the review of literature on NIRS findings before and after treatment.

## Conclusion

A prospective cohort study is recommended to evaluate the utility of two-site NIRS monitoring in the detection of PLCS. NIRS values should be interpreted as trends along with echocardiographic findings to guide goal directed interventions.

## Ethics Statement

Written informed consent was obtained from parents of both patient for the publication of any potentially identifiable images or data included in this article.

## Author Contributions

All authors listed have made a substantial, direct and intellectual contribution to the work, and approved it for publication.

## Conflict of Interest

The authors declare that the research was conducted in the absence of any commercial or financial relationships that could be construed as a potential conflict of interest.
