# Supplementary material for: Findings From Somatic and Cerebral Near-Infrared Spectroscopy and Echocardiographic Monitoring During Ductus Arteriosus Ligation: Description of Two Cases and Review of Literature
Source: Front Pediatr. 2020 Sep 2;8:523. doi: 10.3389/fped.2020.00523 (PMC7492561; doi:10.3389/fped.2020.00523)
Supplement: Supplementary file 1 [file Table_1.DOCX]

| **Author, year** | **Population** | **NIRS measurements** | **Study objective** | **PDA Intervention** | **NIRS measurement comparison** | | **Other measurements** | **Study Conclusion** | **Observations** |
| --- | --- | --- | --- | --- | --- | --- | --- | --- | --- |
|  |  |  |  |  | Before treatment (mean) | After treatment  (mean) |  |  |  |
| **PHARMACOLOGICAL TREATMENT** | | | | | | | | | |
| Underwood et al, 2007 (11) | n=19 infants (13 treated, 6 not treated)  GA, mean + SD (g): 26 + 0.4 (treated)  28 + 0.9 (not treated).  Birth weight, mean + SD: 841 + 40 (treated)  916 + 22 (not treated). | Lung, brain, skeletal muscle, kidney, within the first 4 days of life within 2 h before or after echocardiogram.  ODISsey tissue oximeter, ViOptix. | Investigate the utility of NIRS in identifying infants who would benefit from early echocardiography. | Indomethacin /Surgical ligation (13),  No treatment (6) . | rScO2: 46 +15  rSrO2: 36+ 16  rSO2 D: 37 +15  rSO2 RL: 49  + 11  rSO2 LL: 47  + 13 | rScO2: 40 + 17  rSrO2: 53 + 13  p = 0.05  rSO2 D: 42 + 14  rSO2 RL : 39 +10  rSO2 LL: 41  + 11 | rSO2 of skeletal muscle and kidney differed between infants who were treated for PDA and those who were not (p = 0.01). | NIRS shows encouraging efficacy in identifying ELBW infants who were likely to benefit from early echocardiography and subsequent intervention to close PDA. | No significant difference between pretreatment (46%) and posttreatment (40%) rScO2.  rSrO2 increased after indomethacin treatment ( p = 0.05). |
| Lemmers et al, 2008 (12) | n= 40 (20 in PDA group, 20 in control group).  GA, mean + SD (g): 28.6 + 1.5 (PDA)  28.5 + 1.5 (control).  Birth weight, mean + SD: 1154 + 268 (PDA)  1055 + 216 (control).  Mean postnatal age at diagnosis: 41 h (17-76). | Cerebral NIRS measured in ten-minute periods during PDA, at 10, 20, 30, 60 and 120 minutes, and at 6, 12, 24 and 36 h after starting indomethacin treatment.  INVOS 4100 Somanetics CORP | Investigate the impact of PDA and its treatment with indomethacin on regional cerebral oxygenation saturation and fractional tissue oxygen extraction. | Indomethacin (20) | rScO2: 62 + 9 | rScO2 : ≈ 65 + 10 | rScO2 significantly lower and FTOE significantly higher compared with control infants. Normalized to control values after 24 h.  *HsPDA: La/Ao ratio > 1.4, internal ductal diameter > 1.4 mm/kg, left pulmonary artery end diastolic flow > 0.2. | A HsPDA has a negative effect on cerebral oxygenation in the premature infant. Subsequent and adequate treatment of PDA may prevent diminished cerebral perfusion and subsequent decreased oxygen delivery. | No significant difference between overall mean cerebral NIRS value pretreatment (62%) and posttreatment (65%) at 36 h.  Blood pressure support was more needed in infants with PDA as compared with control infants (p < .05) |
| Dani et al, 2018 (13) | n = 21 (Paracetamol group 11, Ibuprofen group 10).  GA, mean + SD (g): 27.6 + 1.1 (Paracetamol)  28 + 1.3  (Ibuprofen).  Birth weight, mean + SD: 906 + 147 (Paracetamol)  1045 + 292 (Ibuprofen).  Mean postnatal age at diagnosis: 24-72 h. | Cerebral oxygenation and FTOE were recorded 30 min before and 60 (T1), 180 (T2), 360 (T3) min after beginning of drug infusion. . | Evaluate the possible effects of paracetamol on cerebral oxygenation and cerebral blood flow velocity. | Paracetamol (11)  Ibuprofen (10) | Paracetamol  rScO2: 66.2 + 1.4  Ibuprofen  rScO2: 73 + 1.4 | Paracetamol  T1 67.3 + 5.3  T2 67.5 + 3.5  T3 67.0 + 4.9  Ibuprofen  T1 72.0 + 3.5  T2 74.3 + 6.4  T3 74.2 + 1.4 | Mean flow velocity and resistance index measured with Doppler ultrasound recorded at the same times. | Treatment of HsPDA with paracetamol does not affect cerebral oxygenation in very preterm infants. No differences in rScO2 in infants treated with paracetamol or ibuprofen. | No significant difference between mean cerebral NIRS value pretreatment (66.2%) and posttreatment (67%) at 360 min.  No significant difference between groups. |
| Arman et al, 2020  (14) | n=31 infants with HsPDA.  GA, mean + SD (g): 27.34 + 2.2  Birth weight, mean + SD: 909.7 + 236.  PNA: Enrolled 24-48 h after birth. | Cerebral, renal and mesenteric oxygenations recorded 60 min before treatment and continued until the end of second measurements.  INVOS 5100; Covidien Somanetics. | To determine if NIRS may be used in concordance with Doppler USG to provide additional data for assessment of organ blood flow in preterm infants with HsPDA. | Ibuprofen  (10 mg/kg/day - 5 mg/kg/day –  5 mg/kg/day -) | rScO2: 54.4 +11.2  rSrO2: 35.6 + 11.2  rSmO2: 36.2 + 8.5 | rScO2: 59 + 8.3 (p .016)  rSrO2: 47.2 + 8.1. (p <.001)  rSmO2: 46.8 + 10  (p <.001) | Doppler measurements performed 1 h before the first dose of treatment. Postreatment measurements were performed between 12-24 h after 3 days of treatment.  Posttreatment cerebral, renal and mesenteric FTOE values correlated positively with corresponding RI. | NIRS might be used in monitoring organ blood flow in preterm infants with PDA. | Pretreatment and posttreatment cerebral, renal and mesenteric FTOE values and arterial mean velocities were inversely correlated. |
| **PHARMACOLOGICAL TREATMENT AND SURGICAL LIGATION GROUPS** | | | | | | | | | |
| Chock et al, 2011  (15) | n= 33  GA, mean + SD (w): 27 + 1 (control)  26 + 2 (conservative)  27 + 2 (indomethacin)  26 + 1 (ligation)  Birth weight, mean + SD (g):  899 + 207 (control)  758 + 142 (conservative)  861 + 277 (indomethacin)  841 + 159 (ligation)  Mean postnatal age at treatment:  9+ 7 (conservative)  6 + 6 (indomethacin)  16 + 9 (ligation) | Cerebral NIRS monitoring applied prior to conservative treatment, indomethacin or surgical ligation.  INVOS 5100  Covidien | To compare regional cerebral oxygen saturation (rSO2) as measured by NIRS in VLBW infants with HsPDA treated with conservative management, indomethacin or surgical ligation. | Conservative (7)  Indomethacin (19)  Surgical Ligation (12)  Control (12) | rScO2 control: 69 + 4%  rScO2 conservative: 68 + 9%  rScO2 indomethacin: 68 + 9%  rScO2 ligation: 63 + 13% | rScO2  conservative: 68 + 9%  rScO2 indomethacin: 66 + 9%  rScO2 ligation: 72 + 7% (24 h).  (p = 0.02) | Cranial ultrasound and magnetic resonance imaging data. No associated neuroimaging abnormalities.  *HsPDA: Size (moderate to large), Left to right shunting through ductus or retrograde or absent diastolic aortic flow. | Infants requiring surgical ligation for a HsPDA are at high risk for significant changes in cerebral oxygenation,  whereas those receiving either indomethacin or conservative management maintain relatively stable cerebral oxygenation levels. | There was a significant increase in rScO2 from baseline values in the surgical group (p = 0.02).  No significant difference between pretreatment and posttreatment rScO2 values in conservative and indomethacin group. |
| **SURGICAL LIGATION** | | | | | | | | | |
| Zaramella et al, 2006  (16) | n= 16  GA, mean: 27.3 w (24.34).  Birth weight, mean: 1035.6 (680-1740).  PNA, mean: 16.3 d. (7-33). | Cerebral oxygen saturation was obtained 35 m before ligation and around 14^th^ and 27^th^ min after clip’s insertion.  NIRO 300,  Hamamatsu Photonics | Investigate the effects of PDA ligature on cerebral oxygen saturation, cerebral blood volume and cerebral blood flow velocity by means of NIRS and transcranial Doppler simultaneous examinations. | Surgical ligation after indomethacin or ibuprofen | rScO2: 61.1 (3.8) | rScO2: 56.6 (3.3) 14 min after clip  p = 0.006  rScO2: 55.8 (2.6) 27 min after clip  p = 0.03 | Cerebral blood volume before and after clipping was unvaried.  *Large PDA: 6 newborn (Diameter equating to all or 2/3 of the DA)..  Medium PDA: 8 newborn (Half the diameter of the DA).  Small PDA: 2 newborn  (1/3 the diameter of DA). | A fall in rScO2 suggest an increased oxygen extraction during PDA surgery. The lack of increase in cerebral blood volume or in diastolic flow velocity show that the PDA before clipping did not limited cerebral blood flow. | rScO2 decreased significantly after PDA ligation (61.1 vs 55.8%, p = 0.03) |
| Hüning et al, 2008  (17) | n=10 with HsPDA  GA, mean: 24w (23-27).  Birth weight, mean : 748 g (590-1070).  PNA, mean: 14 d (12-22). | Continued cerebral NIRS recording throughout surgery (40-90 min).  NIRO 300, Hamamatsu Photonics | Examine cerebral blood volume changes occurring after surgical closure of PDA | Surgical ligation after pharmacological treatment (ibuprofen) | rScO2: 53 (15) | rScO2: 47 (22) (0-2 min)  rScO2: 48 (21)  (2-5 min)  rScO2: 51 (22)  (5-10 min). | Changes in cerebral blood volume increased significantly during the first 2 minutes after PDA closure (p = 0.01) and returned to baseline within 2-5 minutes. | There is a short lasting increase in cerebral blood flow immediately after surgical closure of PDA, but no change in cerebral oxygenation. | No significant difference between overall mean cerebral NIRS value preoperatively (53%) and postoperatively (49%). |
| Vanderhaegen et al, 2008  (18) | n=10  GA, mean: 27w (2.6).  Birth weight, mean : 987.5 g (555-1855).  PNA, mean: 33 d (6-88). | Continued cerebral NIRS from 1 h before up to 1 h after clipping.  NIRO 300, Hamamatsu Photonics | Analyze the changes in cerebral tissue oxygenation index (TOI) and fractional tissue oxygen extraction (FTOE) at the time of clipping an after clipping. | Surgical ligation |  | rScO2: increased 2.9% at the exact time of clipping.  p = 0.037  FTOE significantly decreased by 0.02%, (p =0.013) | *Severe PDA: Aortic back-flow, LA/AO > 1.5. | The ductal clipping in se has no negative effect on the cerebral oxygenation. | rScO2: increased significantly (2.9%) at the exact time of clipping, (p = 0.037)  One hour post-clipping rScO2 was not significantly different from control values. |
| MacLaren et al, 2016  (19) | n=11 with HsPDA  GA, mean: 28 w.  Birth weight, mean : 1022 g.  PNA, mean: 29 d. | Cerebral NIRS recorded hourly from 4 h preoperatively until maximum of 24 h post-operatively.  INVOS 5100  Covidien, Ireland. | Review changes in cerebral NIRS preoperatively and postoperatively | Surgical ligation | rScO2: 69%  FTOE: 26% | rScO2: 71 %  FTOE: 24% |  | Cerebral oxygenation is not adversely affected by surgical ligation. | No significant difference between overall mean cerebral NIRS value preoperatively (69%) and postoperatively (71%). |

GA: gestational age PNA: postnatal age SD: Standard deviation rScO2: cerebral tissue oxygenation saturation rSrO2: renal tissue oxygenation saturation rSO2 D: deltoid tissue oxygenation saturation rSO2 RL: Right lung tissue oxygenation saturation rSO2 LL: Left lung tissue oxygenation saturation FTOE: Fractional tissue oxygen extraction HsPDA: Hemodinamically significant patent ductus arteriosus.
